# Acupuncture for Smoking Cessation in Hong Kong: A Prospective Multicenter Observational Study

**DOI:** 10.1155/2016/2865831

**Published:** 2016-11-28

**Authors:** Ying-ying Wang, Zhao Liu, Yuan Wu, Ou Zhang, Min Chen, Ling-ling Huang, Xiu-qing He, Guan-yi Wu, Jin-sheng Yang

**Affiliations:** ^1^Institute of Acupuncture and Moxibustion, China Academy of Chinese Medical Sciences, Beijing, China; ^2^Tobacco Medicine Research and Smoking Cessation Center, China-Japan Friendship Hospital, Beijing, China; ^3^Hong Kong Pok Oi Hospital, Yuen Long, Hong Kong

## Abstract

This was a prospective multicenter observational study, aiming to explore the effects of acupuncture on smoking cessation in Hong Kong. From March of 2010 to August of 2015, a total of 5202 smokers were recruited based on inclusion criteria and treated with acupuncture for 8 weeks. As a result, 2940 subjects finished the study with a drop-out rate of 43.48%. The self-reported 7-day point abstinence rate was 34.00% in Week 8 and 18.40% in Week 52. The exhaled carbon monoxide level and the number of cigarettes smoked per day were reduced significantly after treatment. The time to relapse was calculated to be 38.71 days. In addition, “cigarettes smoked per day,” “Fagerstrom Test for Nicotine Dependence,” “total sessions of acupuncture,” “whether finished 8 acupuncture treatments in the first month,” and “total sessions of acupuncture” were believed to be essential factors for abstinence success. It was concluded that acupuncture was a safe method for smoking cessation and was effective in helping smokers to quit; therefore, acupuncture could be considered as one of the methods to help smokers quit. Further studies regarding the effect differences between acupuncture and medications were needed to clarify the overall benefits of acupuncture.

## 1. Introduction

Smoking is believed to be a serious health threat in the world, which is estimated to involve approximately 6 million deaths each year [1]. As one of the largest countries of tobacco production and consumption [2], China, especially the government of Hong Kong Special Administrative Region (HKSAR) [3], pay full attention to tobacco control. The smoking control in HKSAR was traced back to early 1970s when the first legislation of smoking,* Smoking (Public Health) Regulations*, was established. After then, Hong Kong government started to make a comprehensive tobacco control from aspects of legislative control, tobacco tax, and public education. The latest survey by Census and Statistics Department of Hong Kong in 2013 [4] showed that the current smoking prevalence was at the lowest level during the past 30 years. To further curb the tobacco epidemic, the Hong Kong government has committed to provide the expanded smoking cessation services [5], including hotline of smoking cessation, smoking cessation clinic, and online interactive center of smoking cessation, hoping to provide smoking cessation information, psychological guidance, and medication treatment to smoking quitters.

As one of the essential components of the smoking cessation services, acupuncture is widely accepted in Hong Kong, but its effectiveness is still controversial. The research by Vincet and Richardson [6] showed “whether there is any specific effect of the acupuncture is not yet clear”; a meta-analysis by White et al. [7] believed that “there is no consistent, bias-free evidence that acupuncture has a sustained benefit on smoking cessation for six months or more.” However, the meta-analysis by Castera et al. [8] suggested that “acupuncture may help smokers quit,” while the study by Liu et al. [9] confirmed that the short-term efficacy of acupuncture for smoking cessation was significant.

Therefore, the effect of acupuncture for smoking cessation in Hong Kong was in need of comprehensive evaluation. In order to achieve this propose, with the support from Tobacco Control Office of Health Department of HKSAR as well as State Administration of Traditional Chinese Medicine (TCM) of China, this study was conducted by Institute of Acupuncture and Moxibustion, China Academy of Chinese Medical Sciences, and Hong Kong Pok Oi Hospital. It was hoped to provide references for further application of acupuncture for smoking cessation in Hong Kong.

## 2. Subjects and Methods

### 2.1. Study Design

This was a prospective observational study, which is aimed at preliminarily evaluating the 8-week and 52-week effect of acupuncture for smoking cessation in Hong Kong. This study composed of the preparation period, 8-week acupuncture treatment, and 44-week follow-up visit. The study duration was from March of 2010 to August of 2015. The study sites included 5 clinics of Pok Oi Hospital in Tin Shui Wai, Tsing Yi, Taikoo, Huafu, and Cheung Chau as well as Kwun Tong Community Health Center, Ping Shek TCM Community Clinic, and Causeway Bay Acupuncture Center. In addition, 20 TCM mobile clinics were applied to provide smoking cessation for 48 major areas in Hong Kong.

**Figure 1:**
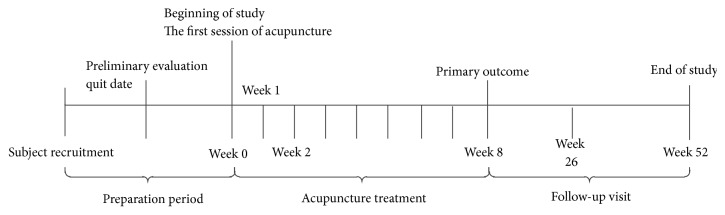
Flow chart of study.

The subjects who met the inclusion criteria were allocated to the study sites to receive acupuncture for smoking cessation. However, control group was not established in this study, because the protocols for sham acupuncture have not been well validated and need to be future studied. The clinical evaluation was performed 1 week, 2 weeks, 8 weeks, 26 weeks, and 52 weeks after treatment.  Figure 1 showed the flow of this study.

### 2.2. Subjects

All the subjects were recruited via local newspaper, hotline of smoking cessation, community events, hospital websites, and recommendation from other medical institutions. Subjects were included into this study if they voluntarily participated in this study; they were daily smokers, aged 18 to 70 years, and were willing to quit smoking; the subjects were excluded if they had mental diseases, serious cardiovascular diseases, apoplexy, or nervous system diseases, had disturbances of blood coagulation, or were pregnant.

The subjects were firstly preliminarily evaluated by researches, and data including personal information, general health condition, and smoking background were recorded. After setting a quit date, subjects were allocated to the study sites. All the subjects signed informed consent before receiving any acupuncture treatment. This study was approved by China Ethics Committee of Registering Clinical Trials (ChiECRCT-2013014).

### 2.3. Acupuncture Procedures

The acupuncture applied in this study was composed of body acupuncture and auricular acupuncture. All the acupuncturists in this study were certified by Chinese Medicine Council of Hong Kong with average acupuncture experience of 5 years; they received guidance and curriculum from Institute of Acupuncture and Moxibustion, China Academy of Chinese Medical Sciences, to guarantee the consistency of acupuncture treatment. Each acupuncture treatment was performed by at least one acupuncturist and one researcher, in which acupuncturist was responsible for the treatment while researcher recorded the Case Report Form to guarantee the accuracy of the study.

#### 2.3.1. Body Acupuncture

*Selection of Acupoints*. Baihui (GV 20), Lieque (LU 7), Hegu (LI 4), Zusanli (ST 36), Sanyinjiao (SP 6), and Taichong (LR 3). Yintang (EX-HN3) was added if there were withdrawal symptoms of cough, running nose, dry eyes, and so forth, while Neiguann (PC 6) was added if there were withdrawal symptoms of dysphoria, melancholy, insomnia, and so forth.

*Body Position*. Subjects were in supine position to expose the body acupoints.

**Figure 2:**
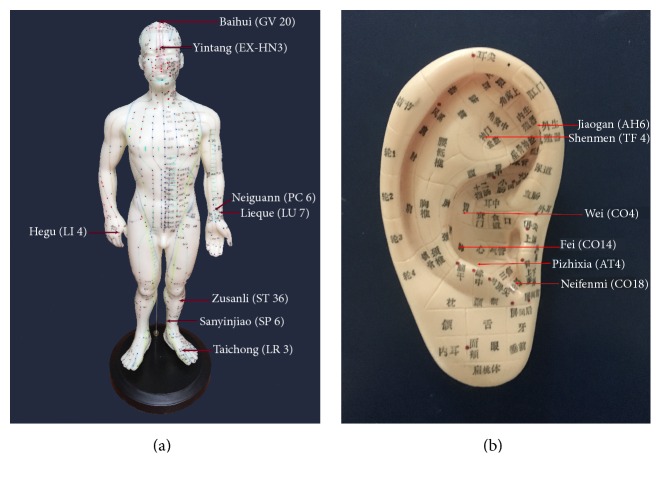
Location of acupoints for smoking cessation ((a) body acupoints and (b) auricular acupoints).

*Acupoint Location*. Refer to the* Acupuncture Point Locations in the Western Pacific Region *by World Health Organization (Figure 2) [10]

*Needle Specification*. 0.25 mm × 40 mm disposable sterile needles were used (purchased from Suzhou Medical Appliance Factory).

*Manipulation of Acupuncture*. Routine fertilization was given on the selected acupoints with 75% medicinal alcohol before acupuncture. Needles were horizontally inserted at Baihui (GV 20) while vertically inserted at other acupoints with a depth of 25 to 50 mm. The mild reinforcing and reducing technique was applied at all acupoints. The arrival of *qi* (needle sensation) was required and needles were required to stay at the acupoints for 30 min. Meanwhile, electroacupuncture device (HuaTuo SDZ-III type) was used and connected at Lieque (LU 7) and Zusanli (ST 36) with continuous wave (15 Hz) for 30 min. The needles were withdrawn quickly to avoid bleeding or hematoma.

*Treatment Frequency*. It was twice per week for 8 weeks.

#### 2.3.2. Auricular Acupuncture

*Selection of Acupoints*. Shenmen (TF_4_), Neifenmi (CO_18_), Pizhixia (AT_4_), Jiaogan (AH_6_), Fei (CO_14_), and Wei (CO_4_) were the acupoints. Kou (CO_1_) was added if there were withdrawal symptoms of nausea, while Zhiqiguan (CO_16_) was added for cough phlegm.

*Body Position*. Subjects were in sitting position to fully expose the auricular acupoints.

*Acupoint Location*. Refer to China National Stand* Nomenclature and Location of Auricular Points* (GB/T 13734-2008) [11].

*Manipulation*. After routine disinfection, the patch of vaccaria seeds with 2 mm in diameter were used and stuck to different auricular acupoints. Each auricular acupoint was pressed for 1 min. In addition, subjects were instructed to press each auricular acupuncture point for 20 s every 1 to 2 hours or whenever they had a craving for smoking.

*Treatment Frequency*. Treatment frequency was twice per week for 8 weeks.

### 2.4. Outcome Measures

The primary outcome was self-reported 8-week 7-day point abstinence rate [12], which was acquired by inquiring the subjects at the 8th week.

The secondary outcomes were self-reported 7-day point abstinence rate in the 1st week, 2nd week, 26th week, and 52nd week, number of cigarettes smoked per day, and exhaled breath carbon monoxide level (device purchased from Bedfont Scientific Company, Maidstone, UK). All the results were acquired by inquiring and testing subjects at each visiting point.

Besides, the safety of acupuncture was assessed. The blood clotting was tested before acupuncture, while blood pressure, pulse, breath, and body temperature were measured before and after treatment as well as in follow-up visit; adverse events were recorded during the study, including the symptoms, occurrence date, and intervention and its relationship with treatment.

### 2.5. Statistical Analysis

According to our plot study [13], the possible abstinence rate of acupuncture for smoking cessation was 20.0%; it was calculated that the sample size necessary to ensure the statistical significance was 784 cases, so we decided to recruit subjects no less than 800 cases.

Intent to Treat (ITT) was applied in this study. SPSS 19.0 statistical software was used for statistical analysis. The measurement data were represented with means ± standard deviation ($\overset{-}{x}\pm s$). *t*-test was used for comparison which met Gaussian distribution and homogeneity of variance, while nonparametric test was used for comparison which did not met homogeneity of variance. Chi-square test was used for numeration data. The Kaplan-Meier curve was used for analyses of time to relapse. The logistic regression analysis was used to calculate the relationship between possible influence factors and abstinence results, represented with OR value and 95% credibility interval. *P* < 0.05 was taken as statistical significance. The authors had no access to information that could identify individual participants during or after data collection.

## 3. Results

### 3.1. Study Process and Baseline Data of the Subjects

**Figure 3:**
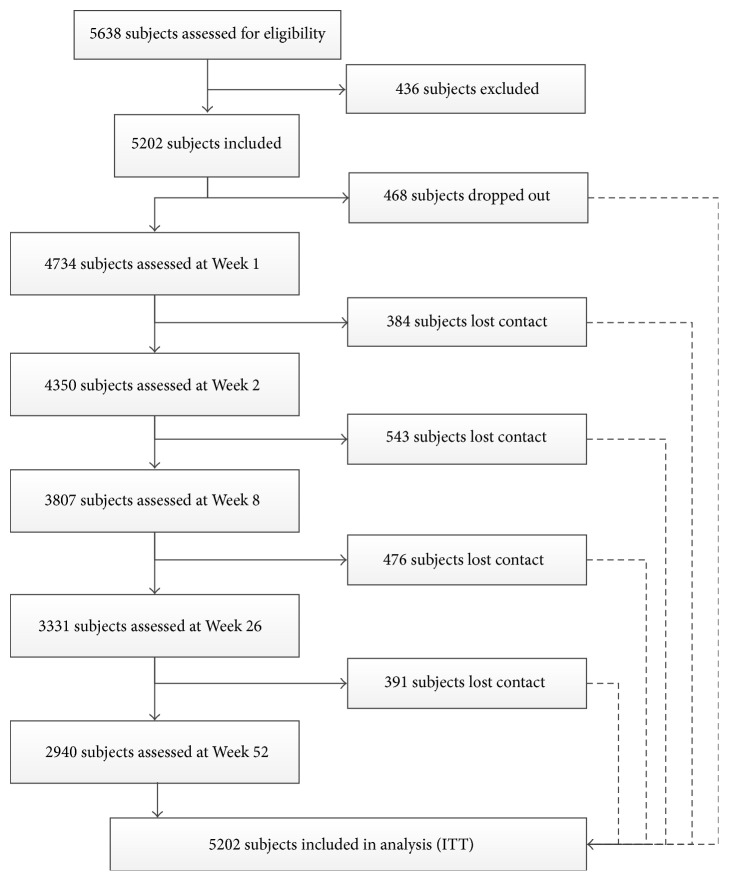
Flow chart for the subjects.

A total of 5638 subjects were recruited. After a preliminary evaluation, 5202 subjects were included into the study. The inclusion for the first subject was on April 7, 2010, while the last follow-up visit was on July 21, 2015. Finally, 2940 subjects finished the study with a drop-out rate of 43.48%.  Figure 3 shows the flow chart of the subjects.

As was shown in Table 1, 5202 subjects who were included in the analysis were characterized with male gender (65.38%), middle age (43.57 ± 12.03 years old), and middle school as primary education level (62.38%). The average smoking duration was (25.05 ± 11.68) years, number of cigarettes smoked per day was (17.67 ± 7.96), Fagerstrom Test for Nicotine Dependence (FTND) was (5.34 ± 2.29), and exhaled breath carbon monoxide level was (15.38 ± 9.82) ppm. The confidence index was (7.37 ± 1.88) and preparation index was (8.11 ± 1.73), indicating strong intention to smoking cessation.

### 3.2. Safety of Acupuncture for Smoking Cessation

During the study, only one case of fainting during acupuncture and one case of hematoma were observed. After intervention, the symptoms were relived and the subjects did not quit the study.

### 3.3. Effects of Acupuncture for Smoking Cessation

The average number of acupuncture received was (5.29 ± 3.24); 2605 subjects finished 8 acupuncture treatments in the first month, while 2721 subjects finished at least 8 treatments in the study.

**Figure 4:**
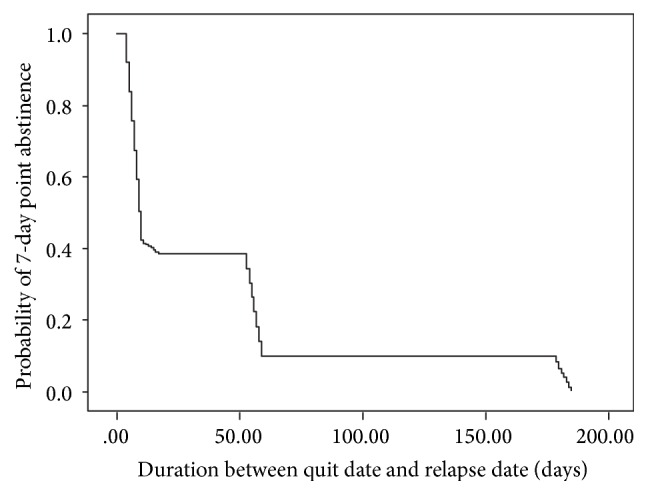
7-day abstinence rate of acupuncture for smoking cessation.

According to the self-reported 7-day point abstinence rate, 1106 subjects succeeded in smoking cessation in the 1st week, 1237 subjects succeeded in the 2nd week, 1769 subjects succeeded in the 8th week, 1142 subjects succeeded in the 26th week, and 955 subjects succeeded in the 52nd week (Figure 4). This indicated that the abstinence rate was increased along with acupuncture treatment but was decreased during the follow-up visit.

As was shown in Table 2, compared to before treatment, the exhaled breath carbon monoxide level was reduced significantly from (15.38 ± 9.82) ppm before treatment to (6.99 ± 6.27) ppm after 8-week treatment (*P* < 0.05); the number of cigarettes smoked per day was also significantly decreased from (17.67 ± 7.96) before treatment to (4.34 ± 5.43) after 8-week treatment (*P* < 0.05) but was significantly increased in follow-up (*P* < 0.05).

**Figure 5:**
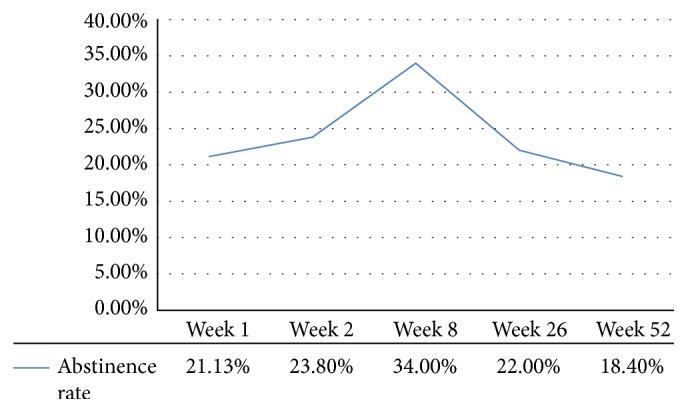
Kaplan-Meier analysis of time to relapse.

The results of Kaplan-Meier analysis showed the time to relapse was 38.71 days (95% CI 37.12–40.32) for acupuncture (Figure 5).

### 3.4. Logistic Regression Analysis of Acupuncture for Smoking Cessation

The relationship between baseline characteristics and study outcomes was calculated by logistic regression analysis. As was shown in Table 3, the 8-week abstinence rate was significantly related to “cigarettes smoked per day,” “FTND,” “total sessions of acupuncture,” and “whether finished 8 acupuncture treatments in the first month,” while the 52-week abstinence rate was significantly related to “cigarettes smoked per day,” “FTND,” and “total sessions of acupuncture.”

## 4. Discussion

This study was aimed at exploring the effects of acupuncture for smoking cessation in HKSAR. It was indicated that 8-week acupuncture was safe for smoking cessation; the abstinence rate was 34.0% in Week 8 and 18.4% in Week 52, respectively. In addition, the exhaled breath CO level and number of cigarettes smoked per day were significantly reduced in subjects who failed in smoking cessation; the time to relapse was 38.71 days, which was longer than 35 days in E-cigarette and 14 days in nicotine patch [14].

This result was consistent with conclusions of 27.15% by Wu et al. [15] and was higher than 12.9% by Fritz et al. [16], 4.1% by Aycicegi-Dinn and Dinn [17], and 5% by Zhang et al. [18]. The inconsistence of conclusions was probably because although these studies claimed their treatment as acupuncture, their acupuncture settings were different, such as acupuncture forms (needle type and electrical stimulation), selection of acupoints, technical skills, and treatment courses, which could affect the clinical effects of acupuncture and result in the inconsistence.

The strengths of our study included rigorous study design and the use of a conservative outcome measure and to minimize the risk of bias.

Firstly, as was demonstrated, a high drop-out rate in smoking cessation study was inevitable, even if the adherence to treatment of different research designs was not satisfactory (19% to 62%) [19]. So we established a large sample size and hoped to ensure the reliability of clinical results as much as possible; according to the recommendation [20], one-year follow-up visit was designed to verify the long-term curative effects of acupuncture for smoking cessation. We hoped the large sample and long-term observation of our study design could provide references of methodology for further study.

Secondly, although several researches [21–23] recommended the expired CO-verified 24-hour point abstinence rate as primary outcome measure to ensure the objectivity and correctness, we noted that failed subjects could be considered as successful by CO-verified 24-hour point abstinence rate if they kept nonsmoking status within 24 hours before evaluation and smoked before and after that, which overrated the curative effects of acupuncture. In this study, self-reported 7-day point abstinence rate combined with verification of expired CO could reflect the effects of acupuncture more correctly.

Thirdly, our acupuncture plan was based on literature research [13] and pilot study [24], aiming to relieve the withdrawal syndromes. For example, in the theory of TCM, Lieque (LU 7) was the connecting point of lung meridian, which could regulate lung* qi*; acupuncture at Zusanli (ST 36) can regulate* qi* movement of spleen and stomach to relieve the discomfort caused by smoking cessation; acupuncture at Baihui (GV 20) can calm the mind to relieve the anxiety. The use of auricular acupoints can not only enhance the effects of body acupoints but also induce subjects to have a negative taste and smell to tobacco. Consequently, the desire for cigarettes was reduced [25]. Due to its effectiveness and convenience, the electroacupuncture device was widely used in acupuncture treatment. For this study was a model for real world interventions; the electroacupuncture device was used at Lieque (LU 7) and Zusanli (ST 36) which were probably the most essential acupoints for smoking cessation.

Fourthly, besides the unified treatment plan, more individual acupuncture for smoking cessation was provided. For example, in order to relieve withdrawal syndrome during the smoking cessation, subjects were instructed to press each auricular acupoint at home or when they felt a strong craving for smoking, especially after dinner or intellectual work. Furthermore, acupuncturists, after took course provided by researchers, were asked to add or reduce acupoints according to different withdrawal symptoms.

Fifthly, a total of 20 TCM mobile clinics for smoking cessation were introduced for subjects who had busy work schedule or lived in the outskirts. Unfortunately, a high drop-out was still inevitable, indicating that the effects of acupuncture on smoking cessation may be related to personal environment and living factors [26]. It was worthy of more attention in future studies. What is more, logistic regression analysis showed “total sessions of acupuncture” was significantly related with 8-week result and 52-week result. Therefore, sufficient acupuncture, particularly at the beginning of smoking cessation, played an essential role in smoking cessation.

Our study had several limitations. Firstly, it was difficult to design an appropriate control group for acupuncture. Sham acupuncture is often used as control, but the protocols for sham acupuncture have not been well validated and need to be future studied [27]. Secondly, the frequency of acupuncture treatment (twice per week) is less than traditional acupuncture theory, which may have negative effects on curative effects. Finally, quite a lot of subjects did not receive sufficient acupuncture because of busy life, job mobility, or lack of acupuncture knowledge. Therefore, it was suggested that public education regarding acupuncture for smoking cessation should be prompted, such as providing community lessons, sending publicity materials of acupuncture, and explaining the theories and advantages of acupuncture. Moreover, successful cases can be introduced so that more people can have a clear understanding about smoking cessation with acupuncture.

## 5. Conclusion

We conclude that acupuncture is a safe method for smoking cessation and is effective in helping smokers to quit; therefore, acupuncture could be considered as one of the methods to help smokers quit in Hong Kong. Further studies regarding the effectiveness differences between acupuncture and nicotine replace treatment, Varenicline, and Bupropion are needed to establish the overall benefits of acupuncture.

## Figures and Tables

**Table 1:** Baseline characteristics of subjects.

Baseline data			*n* (%)
Demographical data	Gender	Male	3401 (65.38%)
		Female	1801 (34.62%)
	Age (years old)		43.57 ± 12.03
	Education background	Below primary school	209 (4.02%)
		Primary school	451 (8.67%)
		Middle school	3245 (62.38%)
		Preparatory course	635 (12.21%)
		College or above	662 (12.73%)

Smoking background	Smoking duration (years)		25.05 ± 11.68
	Number of cigarettes smoked per day		17.67 ± 7.96
	FTND		5.34 ± 2.29
	Previous attempts for smoking cessation	0 times	994 (19.11%)
		1 time	1530 (29.41%)
		2–5 times	2338 (44.94%)
		6–10 times	201 (3.86%)
		10 times or above	139 (2.67%)
	Exhaled breath carbon monoxide level (ppm)		15.38 ± 9.82
	Importance index (0 to 10 points)		8.80 ± 1.46
	Confidence index (0 to 10 points)		7.37 ± 1.88
	Preparation degree (0 to 10 points)		8.11 ± 1.73
	Reason to select acupuncture of traditional Chinese medicine	Advertisement	2630 (50.56%)
		Tried before	259 (4.98%)
		Try new method	1503 (28.89%)
		Believe in acupuncture	810 (15.57%)

**Table 2:** Effects of acupuncture on exhaled breath CO and number of cigarettes smoked per day.

Outcome measures	Before	1st week	2nd week	8th week	26th week	52nd week
Exhaled breath carbon monoxide	15.38 ± 9.82	8.79 ± 7.89	7.55 ± 6.65	6.99 ± 6.27	6.23 ± 6.79	5.45 ± 5.77
Number of cigarettes smoked per day	17.67 ± 7.96	6.23 ± 6.31	4.86 ± 5.47	4.34 ± 5.43	8.82 ± 7.73	10.11 ± 7.48

**Table 3:** Logistic regression analysis of acupuncture for smoking cessation.

Factor type	Influence factor	8 weeks		52 weeks	
		*P* value	OR (95% CI)	*P* value	OR (95% CI)
Demographical data	Gender	0.21	1.14 (0.92–1.42)	0.64	0.92 (0.65–1.31)
	Age	0.50	0.99 (0.98–1.01)	0.65	1.01 (0.98–1.04)
	Education background	0.18	0.92 (0.82–1.04)	0.82	1.02 (0.84–1.23)

Smoking background	Smoking year	0.72	1.00 (0.98–1.02)	0.24	1.02 (0.99–1.05)
	Cigarettes smoked per day	**0.00**	0.97 (0.95–0.99)	**0.00**	1.08 (1.05–1.11)
	FTND	**0.00**	0.55 (0.52–0.59)	**0.00**	3.21 (2.82–3.66)
	Previous attempts of smoking cessation	0.32	6.47 (5.40–7.75)	0.53	1.20 (0.68–2.11)

Reason and intentions to smoking cessation	Reason to choose acupuncture	0.82	0.99 (0.90–1.09)	0.87	0.99 (0.84–1.16)
	Importance index	0.05	1.08 (1.00–1.15)	0.87	0.99 (0.88–1.11)
	Confidence index	0.05	1.07 (1.00–1.16)	0.68	1.03 (0.91–1.15)
	Preparation index	0.88	0.99 (0.92–1.08)	0.86	1.01 (0.88–1.16)

Treatment condition	Total sessions of acupuncture	**0.01**	1.05 (1.01–1.09)	**0.00**	0.57 (0.51–0.62)
	Whether finished 8 acupuncture treatments in the first month	**0.00**	0.45 (0.21–0.97)	0.07	1.96 (0.95–4.02)
	Whether finished 8 acupuncture treatments	0.24	0.04 (0.02–0.09)	0.10	0.97 (0.45–2.10)
